# Long-term Impact of Oral Azithromycin Taken by Gambian Women During Labor on Prevalence and Antibiotic Susceptibility of *Streptococcus pneumoniae* and *Staphylococcus aureus* in Their Infants: Follow-up of a Randomized Clinical Trial

**DOI:** 10.1093/cid/ciy254

**Published:** 2018-03-28

**Authors:** Abdoulie Bojang, Bully Camara, Isatou Jagne Cox, Claire Oluwalana, Kodou Lette, Effua Usuf, Christian Bottomley, Benjamin P Howden, Umberto D’Alessandro, Anna Roca

**Affiliations:** 1 Medical Research Council Unit The Gambia at London School of Hygiene and Tropical Medicine, Fajara; 2 Faculty of Infectious and Tropical Diseases, London School of Hygiene and Tropical Medicine, United Kingdom; 3 Medical Research Council Tropical Epidemiology Group, London School of Hygiene and Tropical Medicine, United Kingdom; 4 Microbiological Diagnostic Unit Public Health Laboratory, Department of Microbiology and Immunology, University of Melbourne, Doherty Institute for Infection and Immunity, Victoria, Australia; 5 Institute of Tropical Medicine, Antwerp, Belgium; 6 Faculty of Epidemiology and Population Health, London School of Hygiene and Tropical Medicine, United Kingdom

**Keywords:** azithromycin, *S. aureus*, *S. pneumoniae*, resistance, West Africa

## Abstract

**Background:**

Oral azithromycin given to women in labor decreases maternal and neonatal bacterial carriage but increases azithromycin-resistant bacteria during at least 4 weeks following the intervention. We assessed the prevalence of bacterial carriage and azithromycin resistance 12 months after treatment among study infants.

**Methods:**

Nasopharyngeal swabs (NPSs) were collected between November 2014 and May 2015 from children aged 11–13 months whose mothers had received azithromycin or placebo during labor. *Streptococcus pneumoniae* and *Staphylococcus aureus* were isolated using conventional microbiological methods. Antibiotic susceptibility was determined by disk diffusion and confirmed by Etest or VITEK-2.

**Results:**

NPSs were collected from 461 children. The prevalence of *S. pneumoniae* and *S. aureus* was similar between children from the azithromycin and placebo arms (85.0% vs 82.1%; odds ratio [OR], 1.23 [95% confidence interval {CI}, .73–2.08] for *S. pneumoniae* and 21.7% vs 21.3%; OR, 1.02 [95% CI, .64–1.64] for *S. aureus*). Prevalence of azithromycin-resistant *S. pneumoniae* was similar in both arms (1.8% vs 0.9% in children from the azithromycin and placebo arms, respectively; OR, 2.10 [95% CI, .30–23.38]); resistance to other antibiotics was also similar between arms. For *S. aureus*, there was no difference in azithromycin resistance between children in the azithromycin (3.1%) and placebo (2.6%) arms (OR, 1.22 [95% CI, .35–4.47]) or resistance to any other antibiotics.

**Conclusions:**

The higher prevalence of *S. aureus* azithromycin resistance observed among women treated during labor and their babies 4 weeks after treatment had waned 12 months after delivery. Azithromycin intervention did not induce other antibiotic resistance to *S. pneumoniae* or *S. aureus*.

**Clinical Trials Registration:**

NCT01800942.

## Teaser

Giving oral azithromycin during labor (2 g) increased prevalence of maternal and neonatal carriage of azithromycin-resistant *Staphylococcus aureus*. Twelve months later, azithromycin resistance had returned to baseline values. Azithromycin resistance in *Streptococcus pneumoniae* was also similar between children from both arms.

Azithromycin is a second-generation broad-spectrum macrolide used to treat infections such as pneumonia, middle ear infections, and sexually transmitted infections [1, 2]. Azithromycin has also been used in mass drug administration (MDA) campaigns to control trachoma in several endemic countries in Africa [3–6]. The impact of these MDA campaigns has varied from one country to another [6–8], but when the baseline prevalence of trachoma is low, 1 round of MDA with azithromycin (MDA-Z) is sufficient to eliminate trachoma [3]. Furthermore, MDA-Z may have beneficial effects beyond trachoma control. In rural Gambia, 3 annual rounds of MDA-Z reduced by 24.2% asymptomatic pneumococcal carriage for at least 1 month [9] and, in Ethiopia, it reduced by 49% all-cause mortality in children aged 1–9 years [10].

Azithromycin has also been given in combination with sulfadoxine-pyrimethamine (SP) during pregnancy with the aim of reducing the prevalence of low birth weight. In Malawi and Papua New Guinea, babies born to mothers who received azithromycin and SP had a reduced risk of low birth weight [11, 12].

MDA-Z may increase the prevalence of macrolide resistance, even after the administration of a single dose. In The Gambia, MDA-Z resulted in a short-term increase in azithromycin-resistant *Streptococcus pneumoniae* [9], while in Tanzania a single dose of MDA-Z increased prevalence of resistance for >6 months [13]. The prevalence was also high in Ethiopia among children after receipt of several annual doses [14] and declined after azithromycin was discontinued [15].

For *Staphylococcus aureus*, another common bacterial pathogen, there are increasing data on the effect of MDA-Z on resistance. In rural Gambia [3], 3 annual rounds of MDA-Z were associated with a long-term (30 months after the first round MDA-Z) increase in both the prevalence of azithromycin-resistant and inducible macrolide lincosamides and streptogramin B (MLS_B_)–resistant *S. aureus* [16]. In Papua New Guinea, the proportion of azithromycin-resistant *S. aureus* was 5 times higher among pregnant women treated with azithromycin than in those in the control group [17].

In a recent trial in The Gambia, administering 2 g of oral azithromycin during labor reduced significantly maternal and neonatal nasopharyngeal carriage of *S. pneumoniae*, *S. aureus*, and group B *Streptococcus* [6], as well as maternal and neonatal infections [18, 19]. Four weeks after birth, children in the intervention arm had similar prevalence of nasopharyngeal carriage of *S*. *pneumoniae* azithromycin resistance compared to those in the control arm (2.1% vs 2.2%; *P* = 1.000), but higher prevalence of *S. aureus* azithromycin resistance (4.5% vs 16.7%; *P* < .001) [20]. To determine the persistence of azithromycin resistance among study children, we collected nasopharyngeal swabs (NPSs) at their first birthday.

## MATERIALS AND METHODS

### Study Site and Population

Trial participants were recruited from the Bundung Maternal and Child Health Hospital, formerly called Jammeh Foundation for Peace, a government-run health center located in western Gambia that manages on average 4500 deliveries per year [21]. The population covers the main ethnic groups in The Gambia with a high illiteracy rate. The climate of the area is typical of the sub-Sahel region.

### Main Trial

Details of the study protocol have been described elsewhere [21]. In brief, this was a phase 3, double-blind, placebo-controlled trial where 829 pregnant women attending the labor ward in the study health facility were randomized to receive a single oral dose of 2 g of either oral azithromycin or placebo (ratio 1:1). The trial started in April 2013 and lasted 14 months (12-month recruitment period and 2 additional months of follow-up). Study participants (women and their newborns) were monitored for 8 weeks after the intervention. Neonatal NPSs were collected during the first 4 weeks of the follow-up as part of the trial. Prevalence of nasopharyngeal carriage of *S. pneumoniae* was significantly lower during the entire neonatal period among neonates exposed to azithromycin (at day 28: 37.2% vs 24.8%; prevalence ratio [PR], 0.67 [95% confidence interval {CI}, .53–.83]; *P* < .001). Prevalence of carriage of *S. pneumoniae*–resistant strains was low throughout and similar between arms (at day 28: 2.1% vs 2.2%; PR, 1.04 [95% CI, .40–2.75]; *P* = 1.000). For *S. aureus*, prevalence of nasopharyngeal carriage peaked at day 6 and by day 28 was still significantly lower in the azithromycin arm (35.3% vs 25.6%; PR, 0.73 [95% CI, .58–.91]; *P* = .005), whereas prevalence of azithromycin-resistant *S. aureus* carriage peaked at day 28 (4.5% vs 16.7% in the azithromycin and placebo arms; PR, 3.68 [95% CI, 2.19–2.75]; *P* < .001) [20].

### Cross-sectional Survey

Between November 2014 and May 2015, children aged 12 months (range, 11–13 months) whose mother had participated in the main trial were visited at home and had an NPS collected. Consent was obtained by a trained nurse blinded to the treatment allocation.

### Ethical Approval

Both the main trial and the cross-sectional survey (CSS) were approved by the Joint Medical Research Council/The Gambia Government Ethics Committee. Mothers of children included in the CSS signed an additional informed consent.

### Sample Handling

The NPS was collected using calcium alginate (Expotech USA) swab as described previously [21]. In brief, each swab was immediately placed into a vial containing skim milk-tryptone-glucose-glycerol transport medium and then into a cold box before being transported to the laboratory within 8 hours [22]. Upon receipt, the tubes were vortexed for a minimum of 20 seconds before being stored in freezers at –70°C.

### Laboratory Methods

Details of the laboratory methods were described previously [21]. In brief, 50 μL of the sample was dispensed onto gentamicin blood agar (GBA) (CM0331 Oxoid, United Kingdom, plus 5% sheep blood) and mannitol salt agar (MSA) (CM0085 Oxoid, United Kingdom) for selective isolation of *S. pneumoniae* and *S. aureus*, respectively.

#### Streptococcus pneumoniae

After 20–24 hours of incubation at 37°C with 5% carbon dioxide, GBA plates were examined for typical α-hemolytic colonies. Morphologically distinct α-hemolytic colonies were selected and subcultured on another blood agar to obtain pure growth and screened for optochin susceptibility [22]. *Streptococcus pneumoniae* isolates were confirmed as described previously [21].

#### Staphylococcus aureus

Following 48 hours of incubation at 37°C, MSA plates were examined for typical staphylococci colonies. Pale to golden yellow dome-shaped colonies 1–2 mm in diameter were cultured onto blood agar to obtain pure growth. A coagulase test was performed on all suspected colonies using the Staphaurex plus kit (Oxoid, United Kingdom, catalog number OXR30950201). Isolates testing positive for coagulase were confirmed to be *S. aureus.*

### Antibiotic Susceptibility Testing

Antibiotic susceptibility was performed as described previously [21]. In brief, resistance was initially screened by disk diffusion method. Both *S. pneumoniae* and *S. aureus* isolates were screened using the following antibiotic discs: azithromycin (15 μg), chloramphenicol (30 μg), clindamycin (2 μg), and erythromycin (15 μg). In addition, only *S. pneumoniae* isolates were screened for trimethoprim-sulfamethoxazole (1.25/23.75 μg) and oxacillin (1 μg), and *S. aureus* isolates were screened for cefoxitin (30 μg). All isolates that were of intermediate or resistant by the disk diffusion method were further tested using the Etest for confirmation except those isolates resistant to cotrimoxazole. Due to limited resources, only 90 of these isolates were randomly selected to be confirmed by the Etest. Susceptibility to the different antibiotics was determined following the Clinical and Laboratory Standards Institute guidelines [23]. In addition, D-test was performed on all macrolide (azithromycin and/or erythromycin)–resistant clindamycin-sensitive isolates to assess inducible clindamycin resistance [24]. In an additional effort to confirm resistance, all *S. pneumoniae* isolates found to be resistant by Etest to azithromycin, erythromycin, or clindamycin were retested using the VITEK-2 (bioMérieux, France). The VITEK-2 results were considered definitive.

### Data Management and Statistical Analysis

Laboratory data were transcribed onto bar-coded forms and submitted to data management for entry. The data were double entered in OpenClinica (www.openclinica.com), and antibiotic susceptibility data for *S. pneumoniae* were stored in a REDcap database.

For each antibiotic, the resistant bacterial carriage prevalence was compared between trial arms using the χ^2^ test. This was done for both *S. pneumoniae* and *S. aureus*. In addition, we assessed if resistance up to day 28 posttreatment was associated with resistance 12 months later. For this analysis, only children who were sampled during all 5 time points in the trial (days 0, 3, 6, 14, and 28) and were also sampled in the 12-month CSS were included. All analyses were carried out using Stata version 12.0 software.

## RESULTS

### Study Population

Among 814 study children alive at the end of the initial trial period (8 weeks of age), 5 had died and 196 were >13 months at the time of the CSS survey. Among the remaining 613 children, 465 (76.0%) were enrolled in this study and NPSs were collected from 461 of them (99.1%). Demographic characteristics were similar between children included in the CSS and those who were not (Table 1). Baseline characteristics (sex, ethnic group, season at birth, maternal age, and age at follow-up) in the azithromycin (n = 226) and the placebo groups (n = 235) were similar, except for a higher number of multiple pregnancies in the placebo arm (*P* = .037; Table 2).

**Table 1.** Comparison of Children in the 12-Month Survey With Those Who Did Not Participate

Variable	12-Month Survey, no./No. (%)		*P* Value
	Included	Not Included	
Sex			
Female	230/465 (49.5)	175/378 (46.3)	.360
Male	235/465 (50.5)	203/378 (53.7)	
Ethnicity			
Mandinka	201/462 (43.5)	151/361 (41.8)	.367
Wollof	49/462 (10.6)	45/361 (12.5)	
Jola	80/462 (17.3)	47/361 (13.0)	
Fula	76/462 (16.5)	68/361 (18.8)	
Other	56/462 (12.1)	50/361 (13.9)	
Maternal age at delivery, y			
18–19	30/465 (6.5)	37/378 (9.8)	.109
20–29	295/465 (63.4)	244/378 (64.6)	
≥30	140/465 (30.1)	97/378 (25.7)	
Multiple pregnancies			
No	453/465 (97.4)	362/372 (95.8)	.183
Yes	12/465 (2.6)	16/378 (4.2)	

**Table 2.** Baseline Characteristics of Mothers and Children in the 2 Groups

Characteristic	Azithromycin Group (n = 226)	Placebo Group (n = 235)	*P* Value
Maternal age at delivery, mean (SD)	26.5 (5.3)^a^	26.6 (5.0)	.684
Mode of delivery			
Vaginal	220 (97.4)	233 (99.2)	.165
Cesarean	6 (2.7)	2 (0.9)	
Information at birth			
Multiple pregnancy	2 (0.9)	10 (4.3)	.037
Sex^b^			
Female	117 (51.8)	104 (44.3)	.266
Male	107 (47.4)	124 (52.8)	
Ethnicity^c^			
Mandinka	93 (41.2)	106 (45.1)	.490
Jola	44 (19.5)	36 (15.3)	
Wollof	26 (11.5)	23 (9.8)	
Fula	37 (16.4)	38 (16.2)	
Others	24 (10.6)	31 (13.2)	
Season			
Dry	178 (78.8)	188 (80.0)	.909
Wet	48 (21.2)	47 (20.0)	
Child age at follow-up visit, mean (SD)	12.24 (0.6)	12.27 (0.6)	.486

Data are presented as No. (%) unless otherwise indicated.

Abbreviation: SD, standard deviation.

^a^Three missing data in the azithromycin arm.

^b^Nine missing data (2 in the azithromycin arm and 7 in the placebo arm).

^c^Three missing data (2 in the azithromycin arm and 1 in the placebo arm).

### Prevalence of Bacterial Carriage

The prevalence of *S. pneumoniae* carriage was high and similar between arms (85.0% vs 82.1% in the azithromycin and placebo arms, respectively; odds ratio [OR], 1.23 [95% CI, .73–2.08]; Table 3). The prevalence of *S. aureus* carriage was lower but also similar between arms (21.7% vs 21.3% in the azithromycin and placebo arms respectively; OR, 1.02 [95% CI, .64–1.64]).

**Table 3.** Univariate Analysis of Bacterial Carriage by Arm After 12 Months

Bacteria	Azithromycin (n = 226), No. (%)	Placebo (n = 235), No. (%)	OR	(95% CI)	*P* Value
*Streptococcus pneumoniae*					
Yes	192 (85.0)	193 (82.1)	1.23	(.73–2.08)	.413
No	34 (15.0)	42 (17.9)			
*Staphylococcus aureus*					
Yes	49 (21.7)	50 (21.3)	1.02	(.64–1.64)	.916
No	177 (78.3)	185 (78.7)			

Abbreviations: CI, confidence interval; OR, odds ratio.

### Prevalence of Antibiotic Resistance

#### Streptococcus pneumoniae

**Figure 1.**
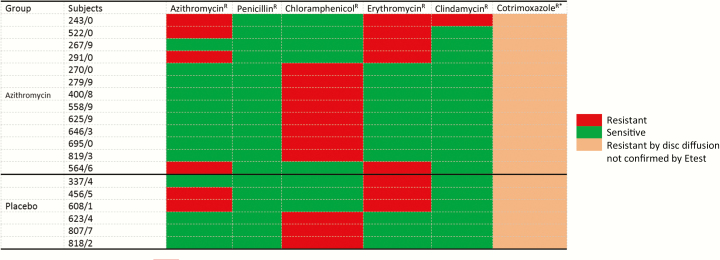
Heat map showing pattern of *Streptococcus pneumoniae* isolates resistant (R) to at least 1 antibiotic (except cotrimoxazole), by arm. Resistance Etest cutoff values: azithromycin, ≥2 μg/mL; penicillin, ≥8 μg/mL; chloramphenicol, ≥8 μg/mL; erythromycin, ≥1 μg/mL; and clindamycin, ≥1 μg/mL. *Cotrimoxazole susceptibility patterns for isolates resistant to at least 1 of azithromycin, penicillin, chloramphenicol, erythromycin, and clindamycin are included in the table. Resistance cutoff value was ≤18 mm using disk diffusion.

Only 6 of the 385 *S. pneumoniae* isolates were resistant to azithromycin, and prevalence was similar between study arms (1.8% vs 0.9% in the azithromycin and placebo arms, respectively; OR, 2.10 [95% CI, .30–23.38]). Prevalence of *S. pneumoniae* resistance against the other antibiotics tested was also similar between arms (Table 4). D-test was performed on 14 samples (6 azithromycin and 8 erythromycin resistant) and all were negative. Resistance to cotrimoxazole based on disk diffusion was the highest among all the antibiotics tested (Table 4). Among the cotrimoxazole-resistant isolates, 30% were retested using Etest and 63.3% (57 of 90) were confirmed. All *S. pneumoniae* isolates were susceptible to at least 3 of the antibiotics tested, except for 1 isolate in the azithromycin arm that was susceptible to only 2 antibiotics (Figure 1).

**Table 4.** Prevalence of Antibiotic-Resistant Bacteria, by Arm, After 12 Months

Bacteria and Antibiotic		Azithromycin (n = 226), No. (%)	Placebo (n = 235), No. (%)	OR	(95% CI)	*P* Value
*Streptococcus pneumoniae*						
Azithromycin^R^	Yes	4 (1.8)	2 (0.9)	2.10	(.30–23.38)	.384
	No	222 (98.2)	233 (99.1)			
Erythromycin^R^	Yes	5 (2.2)	3 (1.3)	1.75	(.34–11.38)	.442
	No	221 (97.8)	232 (98.7)			
Chloramphenicol^R^	Yes	8 (3.5)	3 (1.3)	2.84	(.67–16.78)	.111
	No	218 (96.5)	232 (98.7)			
Penicillin^R^	Yes	0 (0.0)	0 (0.0)	NA		
	No	226 (100.0)	235 (100.0)			
Clindamycin^R^	Yes	1 (0.4)	0 (0.0)	NA		.307
	No	225 (99.6)	235 (100.0)			
Cotrimoxazole^R,a^	Yes	140 (92.1)	152 (93.3)	0.84	(.33–2.17)	.696
	No	12 (7.9)	11 (6.8)			
*Staphylococcus aureus*						
Azithromycin^R,b^	Yes	7 (3.1)	6 (2.6)	1.22	(.35–4.47)	.724
	No	219 (96.9)	229 (97.5)			
Erythromycin^R,b^	Yes	5 (2.2)	5 (2.1)	1.04	(.24–4.59)	.950
	No	221 (97.8)	230 (97.9)			
Cefoxitin^R^	Yes	0 (0.0)	2 (0.9)	NA	(.00–1.99)	.165
	No	226 (100.0)	233 (99.2)			
Chloramphenicol^R^	Yes	1 (0.4)	3 (1.3)	0.34	(.01–4.32)	.334
	No	225 (99.6)	232 (98.7)			
Clindamycin^R^	Yes	6 (2.6)	7 (3.0)	0.89	(.24–3.14)	.834
	No	220 (97.4)	228 (97.0)			

Testing was done with Etest and VITEK-2.

Abbreviations: CI, confidence interval; NA, not applicable; OR, odds ratio; R, resistant.

^a^Only 63.3% (57/90) of cotrimoxazole-resistant *S. pneumoniae* isolates by disk diffusion were confirmed by Etest.

^b^Isolates of intermediate azithromycin (4 µg/mL) or erythromycin (1–4 µg/mL) resistance were considered sensitive.

#### Staphylococcus aureus

**Figure 2.**
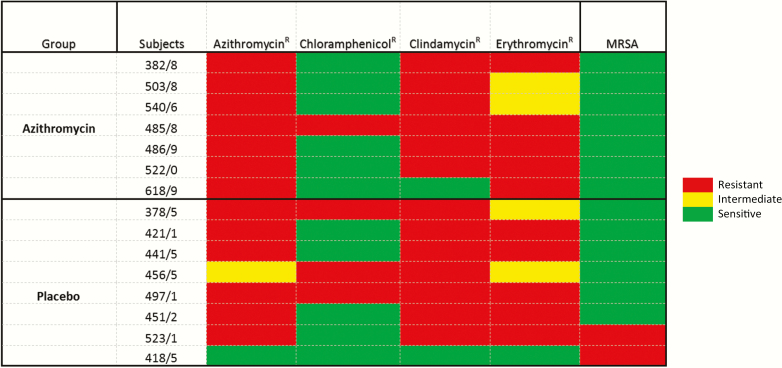
Heat map showing pattern of *Staphylococcus aureus* isolates resistant (R) to at least 1 antibiotic, by arm. Resistance Etest cutoff values: azithromycin, ≥8 μg/mL; chloramphenicol, ≥32 μg/mL; erythromycin, ≥16 μg/mL; clindamycin, ≥4 μg/mL; cefoxitin for methicillin-resistant *Staphylococcus aureus* (MRSA), ≥8. Intermediate Etest cutoff values: azithromycin, 4 μg/mL; erythromycin, 1–4 μg/mL.

Overall, 13 of the 99 *S. aureus* isolates were resistant to azithromycin, and prevalence was similar between study arms (3.1% vs 2.6% in the azithromycin and placebo arms, respectively; OR, 1.22 [95% CI, .35–4.47]). Prevalence of *S. aureus* resistance against the other antibiotics was low (<3.0%) and similar between arms (Table 4). All *S. aureus* isolates from both arms were susceptible to at least 1 of the antibiotics tested (Figure 2).

### Association of Azithromycin Resistance up to 28 Days and 1 Year Posttreatment (CSS)

There were 427 participants with complete information on *S. pneumoniae* and *S. aureus* resistance (samples at the 5 time points during the neonatal period and at 1 year posttreatment). For *S. pneumoniae*, 0.73% of the participants not carrying a resistance strain during the neonatal period had a resistant strain 1 year after birth; prevalence of resistant strains was significantly higher (14.3%) in those carrying a resistant strain during the neonatal period (*P* < .001). For *S. aureus*, prevalence of resistant strains at 1 year was not associated with carriage during the neonatal period (2.6% vs 3.9%; *P* = .524).

## DISCUSSION

Twelve months after administering azithromycin to women in labor, the prevalence of *S. aureus* azithromycin resistance previously observed during the neonatal period had returned to baseline levels, with no differences between study arms. This was also true for *S. pneumoniae* azithromycin resistance.

Prevalence of azithromycin-resistant pneumococcal carriage was low among infants from both groups. This was expected as prevalence of resistance was low and similar between trial arms during the 4 weeks following the intervention [20]. Our results are similar to those of the Gambian trachoma trial in which children received an annual dose of azithromycin for 1 or 3 years [9] and prevalence of *S. pneumoniae* azithromycin resistance was similar between arms 6 months after dose 3. They are also consistent with the trial conducted in northern Tanzania, where no association of prevalence of macrolide-resistant *S. pneumoniae* was found 6 months following MDA-Z [25]. Nevertheless, in some studies azithromycin resistance persists after MDA-Z. In Ethiopia, prevalence of azithromycin-resistant *S. pneumoniae* increased after 4 rounds of MDA-Z for trachoma control and did not return to baseline 12 months after the last round [14]. This is probably due to the higher prevalence of resistance at baseline (9.2%) [14] as compared to The Gambia (1.4%) [20] and Tanzania (0%) [25], and to the larger number of MDA-Z rounds. Indeed, frequent exposure to treatment and higher resistance at baseline are associated with increased prevalence of macrolide resistance that persists for a very long time [26].

Only 1 isolate (from the azithromycin arm) showed resistance to both macrolides and clindamycin, suggesting constitutive resistance. All clindamycin-sensitive, macrolide-resistant isolates had a negative D-test, indicating no evidence of inducible resistance. Macrolide exposure could potentially induce clindamycin resistance through methylation of the common ribosomal binding site for macrolides, clindamycin as well as streptogramin B (MLS_B_), often referred to as the MLS_B_ phenotype [27]. However, in our study population, macrolide exposure did not induce resistance to clindamycin.

For cotrimoxazole, two-thirds of the *S. pneumoniae* isolates resistant by disk diffusion were confirmed by Etest and, therefore, actual resistance was probably around 60%. Such high prevalence among the children has been previously described [28] and is probably the result of high cotrimoxazole use in The Gambia; no increase as a result of the intervention was observed.

Nearly all *S. aureus* isolates resistant to azithromycin were resistant to erythromycin and approximately two-thirds were also resistant to clindamycin, suggesting that the underlying mechanism was constitutive, mediated by the *erm* gene [27]. There were 2 cases of methicillin-resistant *S. aureus* (MRSA) in the study, both in children from the placebo arm, and therefore not associated with the intervention. To our knowledge, this is the first time MRSA has been reported from carriage in The Gambia, although it was previously reported from invasive isolates [29].

This study had some limitations. One is the lack of information between the last sample collection in the main trial and the survey done at the infants’ first birthday. We were unable to determine how long *S. aureus*–resistant isolates persisted. Such information is important to determine the potential risk of resistance transmission and establishment within the population. Despite the lack of information on antibiotic use by the study participants between 2 and 12 months of age, we did not expect major differences between trial arms, as this was a randomized trial. The survey carried out at about 12 months postintervention was able to include about two-thirds of study participants, which is a substantial proportion of the study population; we did not observe significant differences between children included in the survey and those left out. Exposure to a macrolide in our study population may have also resulted in the emergence of macrolide-resistant, gram-negative bacteria. In Tanzania, rectal swabs collected from young children following MDA-Z exposure were significantly associated with higher azithromycin-resistant *Escherichia coli* carriage at 1 month post-MDA (OR, 15.27; *P* < .001) and all subsequent surveys [30]. Our analysis was based on gram-positive bacteria as we only collected NPS.

In conclusion, administering 2 g of azithromycin to Gambian women in labor induced a transient azithromycin resistance in *S. aureus* that lasted <12 months. Although the long-term impact on prevalence of resistance of the 2 bacteria is reassuring, pathogenicity and transmissibility of resistant *S. aureus* strains observed in the short term warrant further investigation.

## Notes

***Financial support.*** This work was supported by the Medical Research Council (MRC) Unit The Gambia PhD fellowship. The main trial was jointly funded by the UK MRC and the UK Department for International Development (DFID) under the MRC/DFID Concordat agreement (reference number MR/J010391/1) and is also part of the EDCTP2 program supported by the European Union.

***Acknowledgments.*** The authors give special thanks to the field team for assisting with sample collection, and to the study participants for agreeing to take part in the study.

***Potential conflicts of interest.*** All authors: No reported conflicts of Interest. All authors have submitted the ICMJE Form for Disclosure of Potential Conflicts of Interest. Conflicts that the editors consider relevant to the content of the manuscript have been disclosed.
